# Lithium is able to minimize olanzapine oxidative-inflammatory induction on macrophage cells

**DOI:** 10.1371/journal.pone.0209223

**Published:** 2019-01-29

**Authors:** Marcelo Soares Fernandes, Fernanda Barbisan, Verônica Farina Azzolin, Pedro Antônio Schmidt do Prado-Lima, Cibele Ferreira Teixeira, Ivo Emílio da Cruz Jung, Charles Elias Assmann, Rogerio Tomasi Riffel, Marta Maria Medeiros Frescura Duarte, Ednea Maia Aguiar- Ribeiro, Ivana Beatrice Mânica da Cruz

**Affiliations:** 1 Pharmacology Graduate Program, Federal University of Santa Maria, Santa Maria, RS,Brazil; 2 Federal University of the Southern Frontier, Passo Fundo, RS, Brazil; 3 Gerontology Graduate Program, Federal University of Santa Maria, Santa Maria, RS, Brazil; 4 BraIn Institute, Catholic University of Rio Grande do Sul, Porto Alegre-RS, Brazil; 5 Biochemical Toxicology Graduate Program, Federal University of Santa Maria, Santa Maria, RS, Brazil; 6 Hospital of Clinics of Passo Fundo, Passo Fundo, RS, Brazil; 7 Lutheran University of Brazil, Santa Maria- RS, Brazil; 8 Open University of the Third Age, State University of Amazonas, Manaus-AM, Brazil; ASCR, Institute of Physics, CZECH REPUBLIC

## Abstract

**Background:**

Olanzapine (OLZ) is a second-generation antipsychotic drug used for treatment of schizophrenia, bipolar disorder, and other neuropsychiatric conditions. Undesirable side effects of OLZ include metabolic alterations associated with chronic oxidative-inflammation events. It is possible that lithium (Li), a mood modulator that exhibits anti-inflammatory properties may attenuate OLZ-induced oxi-inflammatory effects.

**Methodology:**

To test this hypothesis we activated RAW 264.7 immortalized macrophages with OLZ and evaluated oxidation and inflammation at the gene and protein levels. Li and OLZ concentrations were determined using estimated plasma therapeutic concentrations.

**Results:**

OLZ triggered a significant increase in macrophage proliferation at 72 h. Higher levels of oxidative markers and proinflammatory cytokines, such as TNF-α, IL-1β, and IL-6, with a concomitant reduction in IL-10, were observed in OLZ-exposed macrophages. Lithium (Li) exposure triggered a short and attenuated inflammatory response demonstrated by elevation of superoxide anion (SA), reactive oxygen species (ROS), IL-1β, and cellular proliferation followed by elevation of anti-inflammatory IL-10 levels. Li treatment of OLZ-supplemented macrophages was able to reverse elevation of oxidative and inflammatory markers and increase IL-10 levels.

**Conclusions:**

Despite methodological limitations related to *in vitro* protocols, results suggested that Li may attenuate OLZ-induced oxidative and inflammatory responses that result from metabolic side effects associated with OLZ.

## Introduction

Olanzapine (OLZ) is an atypical antipsychotic drug of the thienobenzodiazepine class. OLZ blocks multiple neurotransmitter receptors, including D2 and 5-HT3 receptors. OLZ is used to treat resistant and non-resistant schizophrenia in children and adolescents [1,2], therapeutic maintenance of bipolar disorder [2,3], management of agitation in adults with progressive dementia [4], and treatment of Huntington's Disease [5,6]. Moreover, OLZ is used to attenuate chemotherapy-induced nausea and vomiting, including in children under 13 years of age [7,8].

OLZ has pleiotropic effects by acting on diverse neurotransmission pathways. However, the non-specific nature of this antipsychotic drug results in numerous metabolic side effects including weight gain, dry mouth, somnolence, constipation, and increased appetite. Clinical studies, such as Ferno et al [9], showed dose-dependent lipogenic effects due 5-HT2C and H1 receptor antagonism after the first 6 weeks of treatment. Furthermore, OLZ causes glycemic disruptions associated with increased risk of developing type 2 diabetes mellitus. Continuous use of OLZ has been associated with higher cardiovascular risk [10].

Studies have suggested that chronic inflammatory processes, via macrophages and inflammatory cytokines, may be responsible for increased risk of obesity [11]. In fact, oxidative-inflammatory mechanisms have been hypothesized to play a role in onset of metabolic side effects associated with OLZ administration [12,13]. This OLZ action involves macrophages activation, that is a cell presenting in blood and in tissues such as fat-tissue [14–15]. Exposure of human adipose-derived stem cells to several antipsychotics, including OLZ, resulted in up-regulation on gene expression and levels of pro-inflammatory cytokines such as IL-1β, NF-κβ, and IL-8, suggesting a direct effect on the immune system [13].

Furthermore, administration of OLZ for five weeks resulted in weight gain, increased visceral fat, infiltration of macrophages, and higher TNF-α, IL-1β, and IL-6 levels in rat fat-tissue [15,16]. OLZ was also able to upregulate macrophage migration inhibitory factor in adipose tissue reducing lipolysis and increasing lipogenic pathways [17].

Indeed, exposure of human adipose-derived stem cells to several antipsychotics, including OLZ, resulted in up-regulation on gene expression and levels of pro-inflammatory cytokines such as IL-1β, NF-κβ, and IL-8, suggesting a direct effect on the immune system [13]. Furthermore, administration of OLZ for 5 weeks resulted in weight gain, increased visceral fat, infiltration of macrophages, and increased levels of TNF-α, IL-1β, and IL-6 in rat adipose tissue and hypothalamus [15].

As polypharmacy is a common clinical practice, investigation of whether psychiatric drugs with anti-inflammatory properties may attenuate metabolic side effects of pro-inflammatory drugs when administered simultaneously [18].This is the Lithium (Li) case, that is an efficient mood modulator molecule concomitantly used in management of acute mania or manic episodes associated with bipolar disorder [19] and to treat some mixed episodes of major depressive and bipolar disorders [20]. Moreover, the combination of Li plus OLZ has been indicated to treat some mixed episodes of major depressive and bipolar disorders [2,20]. Previous in vitro study also suggested that Li could modulate *in vitro* proinflammatory macrophage response exposed to some antidepressant drugs [21]. To test this hypothesis, an in vitro study was performed her using RAW 264.7 macrophages exposed at different OLZ and Li at a plasmatic therapeutic range concentration.

## Material and methods

### Chemicals and equipment

All chemicals and reagents used in this study were analytical grade and purchased from Merck (Darmstadt, Germany) or Sigma-Aldrich Co. (St. Louis, MO, USA). All experiments were performed using pure Li, obtained from Sigma- Aldrich (St. Louis, MO, USA). Plastics and reagents used for cell culture procedures were acquired from Gibco (Thermo Fisher Scientific; Grand Island, NY, USA). Molecular biology reagents were as follows: TRIzol reagent (Thermo Fisher Scientific; Grand Island, NY, USA), iScript cDNA synthesis kit (Bio-Rad Laboratories; Hercules, CA, USA), DNase (Invitrogen Life Technologies; Carlsbad, CA, USA), QuantiFast SYBR Green PCR Kit (Qiagen, Hilden, Germany). Immunoassay Kits were purchased from Abcam (Cambridge, MA- USA), Protocols involving spectrophotometric and fluorimetric analysis were executed using a 96-well microplate reader (SpectraMax M2/M2e Multimode Plate Reader; Molecular Devices- Sunnyvale, CA, USA).

### General experimental design

*In vitro* experiments were performed using a commercially available RAW 264.7 immortalized macrophage cell line (ATCC TIB-71) obtained from the American Type Culture Collection (ATCC, Manassas, VA, USA) cultured under standardized conditions. Cells were cultured using Dulbecco’s Modified Eagle Medium (DMEM) with 10% fetal bovine serum (FBS), supplemented with 1% penicillin/streptomycin and 1% amphotericin B antifungal. Cell cultures were maintained at 37°C in a humidified atmosphere of 5% CO_2_ and expanded to obtain cell number to perform the experiments.

All protocols performed were similar to previously described studies by Barbisan et al. [22] and Duarte et al. [23] of *in vitro* psychotropic drug effects on macrophage inflammatory response. Macrophages are highly sensitive to antigens and other antigenic factors, which induce intense cellular proliferation and morphological changes. These alterations, especially cellular proliferation in 72 h cell cultures, can be used as markers of inflammatory activation of immune cells. A concentration-effect curve (0.007 to 1000 μg/mL) was generated using OLZ concentrations bracketing plasma therapeutic (0.02 and 0.08 μg/mL) OLZ values. OLZ plasma therapeutic values are published in guidelines for therapeutic drug monitoring in neuropsychopharmacology [24]. Phytohemagglutinin (PHA) antigen (125 μg/mL) treatment was used as a positive inflammatory control [21].

The OLZ concentration that triggered the highest inflammatory response was chosen and confirmed by evaluation of cytomorphological alteration, cell cycle modulation, and protein and gene expression analysis of pro- and anti-inflammatory cytokines. Following determination of OLZ concentration, macrophages were concomitantly exposed to OLZ plus Li at 0.7 mEq/L. This concentration was chosen based on Barbisan et al [21].

### Cell proliferation analysis

MTT (3-(4,5-dimethylthiazol-2-yl)-2,5-diphenyltetrazolium bromide) tetrazolium reduction assay was used as a method to evaluate cell proliferation in 72 h cell cultures. This procedure was modified, but we used Mosmann [25] for guidance. Briefly, cells were seeded in 96-well plates at a final concentration of 2×10^5^ cells/mL and MTT reagent was added to the cells at a final concentration of 0.5 mg/mL. After 1 h of incubation at 37°C, formazan crystals were solubilized using dimethyl sulfoxide (DMSO) and absorbance was recorded at 560 nm using a plate reading spectrophotometer (SpectraMax i3x Multi-Mode Microplate Reader; Molecular Devices, Sunnyvale, CA, USA).

### Cell cycle analysis

Cell cycle analysis was performed by flow cytometry. In short, cells were seeded in 6-well plates (1 x 10^4^ cells). Following 72 h treatment, cells underwent resuspension in 70% ethanol and were stored overnight at −20°C. After storage, cells were resuspended in 500 μL of staining solution prepared in PBS, which consisted of 50 μg/mL PI, 100 μg/mL RNase, and 0.05% Triton X-100, and incubated at 37°C for 40 min. Cell cycle analysis was performed using a BD Accuri C6 instrument [26].

### Oxidative markers analysis

Modulation of oxidative stress was evaluated in 72 h cell cultures exposed to OLZ and Li by analysis of superoxide, ROS, and nitric oxide levels. Superoxide levels were quantified using a colorimetric assay that produces a formazan salt via reaction with nitroblue tetrazolium (NBT) chloride, following a protocol previously published by Morabito et al. [27]. Briefly, the cells were seeded in a 96-well plate, diluted in 1× PBS, treated with 10 μL of NBT solution (10 mg/mL), homogenized, incubated at 37°C for 3 h, and centrifuged. 75 μL of supernatant was removed, and an equal volume of DMSO was added to each well. After incubation for 20 min at 37°C, 75 μL of the cell suspension was transferred to another 96-well plate, and the absorbance measured at 540 nm.

Total levels of reactive oxygen species (ROS) were quantified in RAW 264.7 cells using the 2,7-dichlorodihydrofluorescein diacetate (DCFH-DA) assay. DCFH-DA is a nonfluorescent chemical that is deacetylated by mitochondrial esterase enzymes to DCFH which reacts with ROS and becomes DCF, a fluorescent molecule. Fluorescence was recorded at an excitation wavelength of 488 nm and an emission wavelength of 525 nm [28]. NO levels were measured after 72 h by the Griess modified method [29]. In short, RAW 264.7 cells were incubated at room temperature with the Griess reagent for 10 min and the absorbance was recorded at 550 nm wavelength.

### Cytokine immunoassays

All analyses were performed in 72 h cell culture samples. Analysis of cytokine levels (IL-1β, IL-6, TNF-α, and IL-10) provides information on activation of inflammatory pathways. Caspase levels (CASP-8, CASP-3 and CASP-1) may indicate activation of the apoptosis pathway. Cytokines levels in cell culture supernatants were measured, according to the manufacturer’s instructions (Abcam, Cambridge, MA- USA).

Briefly, all reagents and working standards were prepared per manufacturer’s instructions prior to adding 50 μL of the assay diluent RD1W to each well. 100 μL of standard control for each sample was added per well, after which the well was covered with an adhesive strip and incubated for 1.5 h at room temperature. Each well was subsequently aspirated and washed twice, for a total of three washes. The antiserum of each molecule analyzed into wells and the plate was covered with a new adhesive strip, and subsequently incubated for 30 min at room temperature. The aspiration/wash step was repeated, and the conjugate (100 μL) was added to each well and incubated for 30 min at room temperature. Each well was again aspirated and washed before adding 100 μL of substrate solution to each well, followed by incubation at room temperature for an additional 20 min. Following incubation, 50 μL of stop solution was added to each well. Optical density was determined within 30 min at 450 nm using a microplate reader.

### Gene expression analysis

Gene expression of cytokines in RAW 264.7 macrophages (pro inflammatory *IL-1β*, *IL-6*, *TNF-α* and anti-inflammatory *IL-10)* was analyzed by quantitative real-time polymerase chain reaction (qRT-PCR) after 24 h of incubation [21]. Briefly, total RNA was isolated with TRIzol and quantified spectrophotometrically at 260 nm wavelength. Reverse transcription was performed using the iScript cDNA synthesis kit. RNA was added to a final concentration of 1 μg/μL with 0.2 μL of DNase. Generation of cDNA was performed using 1 μL of iScript reverse transcriptase and 4 μL of iScript Mix. Quantitative RT-PCR was performed in a total reaction volume of 20 μL with 1 μL of cDNA and 1x QuantiFast SYBR Green PCR Kit in a Rotor-Gene Q instrument (Qiagen, Hilden, Germany). The specific primer pairs of cytokines used in this study were: IL-1β Forward GCGGCATCCAGCTACGAAT and Reverse ACC AGCATCTTCCTCAGCTTGT; IL-6 Forward TACCCCCAGGAGAAGATTCCA and Reverse CCGTCGAGGATGTACCGAATT; TNF-α Forward CAA CGGCATGGATCTCAAAGAC and Reverse TATGGGCTCATACCAGGGTTTG; IL-10 Forward GTGATGCCCCAAGCTGAGA and Reverse TGCTCTTG TTTTCACAGGGAAGA. Beta-actin, a housekeeping gene was used as an internal control. Relative expression was calculated using comparative CT (Citosine-Timine) and was expressed as the fold expression compared to the control.

### Statistical analyses

The results obtained from all in vitro protocols were analyzed using GraphPad *Prism 6* statistical package software. All experiments were performed in independent triplicates. This data treatment protocol is broadly used in the in vitro analysis in order to allow comparison among results obtained from different days or by different laboratories [30]. Outliers were eliminated considering upper and lower values of 2-SD range since indicated some experimental imprecision. All variables were tested for distribution normality using the Shapiro-Wilk method. Data were compared with one-way or two-way analysis of variance followed by Tukey’s post hoc tests. Data are presented as mean ± SD relative to negative control group. The alpha value was *p* ≤ *0*.*05* to indicate the threshold for statistical significance.

## Results

**Fig 1:**
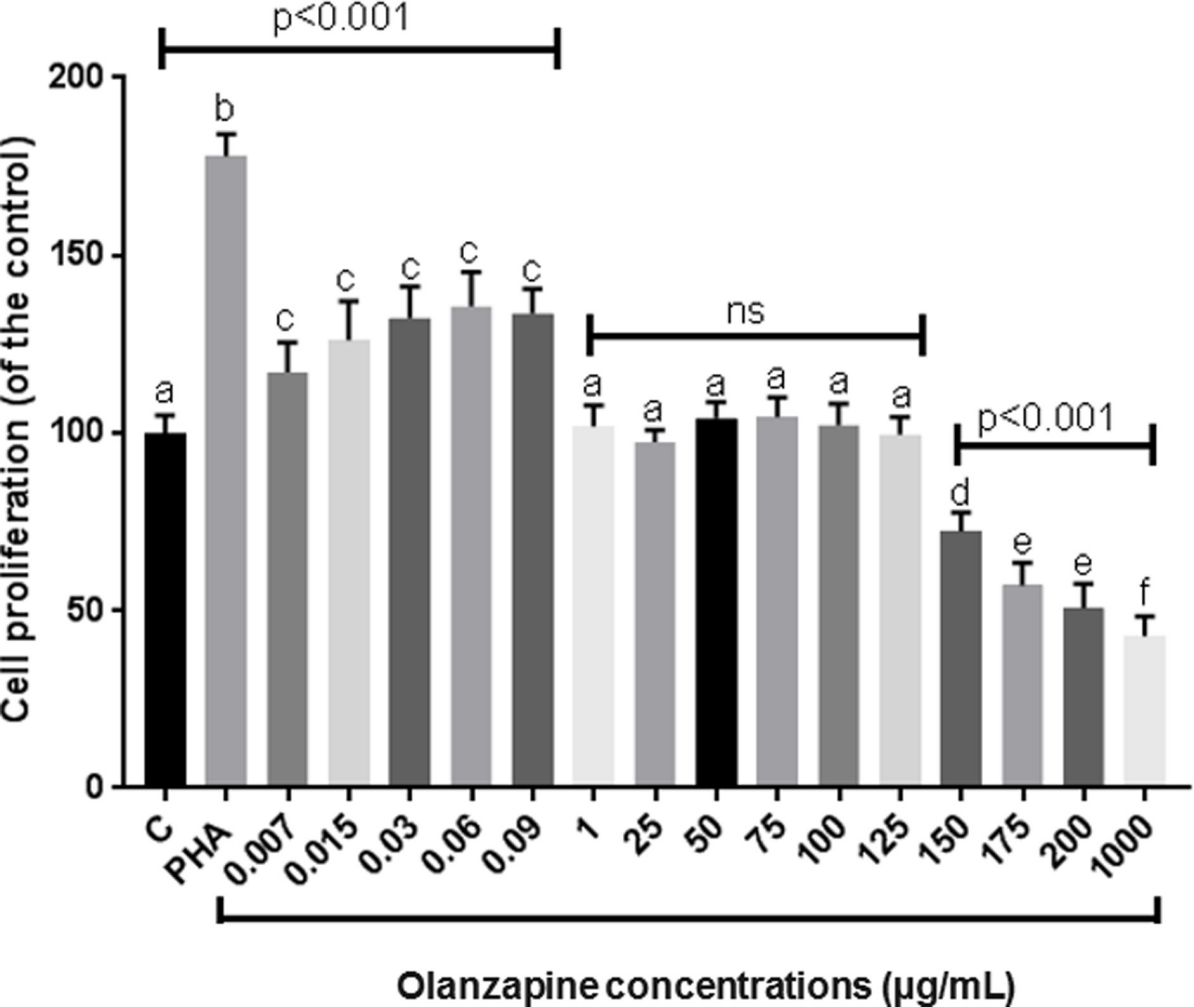
Effect on RAW macrophages cellular proliferation in 72 h cultures e cellular of OLZ (OLZ) at different concentrations. C = non-treated cells; PHA = cells activated by phytohemaglutin antigen exposure. Treatments were compared using one-way analysis of variance (ANOVA), followed by the Tukey *post hoc* test. Different letters (i.e., A, B, C, D, E, F) indicated significant statistical differences among treatments at *p* < 0.05. Treatments with A letter were considered with values similar to C group.

Macrophage proliferation in response to OLZ was evaluated as an indicator of oxi-inflammatory activation (Fig 1). Results showed a hormetic response to OLZ in macrophages, with lower OLZ concentrations (0.007–0.09 μg/mL) resulting in increased cellular proliferation, while higher concentrations significantly decreased growth of cultures, indicating a cytotoxic or immunossupressive OLZ effect (150–1000 μg/mL). The pro-inflammatory effect of OLZ at lower concentrations was confirmed by comparison to a similar response triggered by macrophage exposure to PHA antigen.

**Fig 2:**
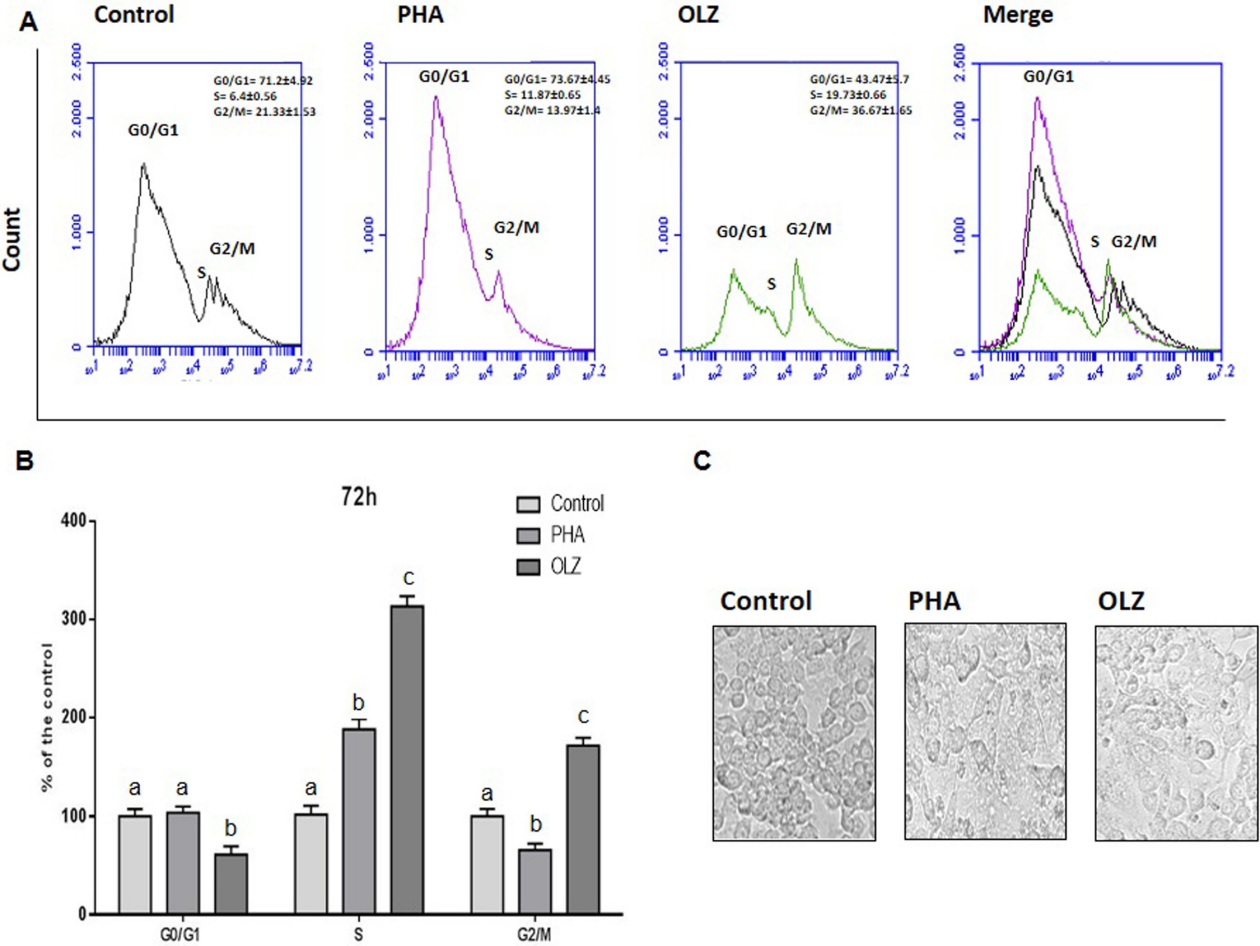
Comparison of cell cycle determined by flow cytometry analysis among RAW macrophages 72 h cultures exposed OLZ (OLZ) at 0.03 μg/mL concentration and to phytohaemagglutinin (PHA), an natural antigen that trigger inflammatory macrophages activation. (A) Representative graphics with cell phases: (G0/G1 = gap 1; S = synthesis; G2/M = gap2 and mitosis). (B) % of cells the control in at each stage (G0/G1 = gap 1; S = synthesis; G2/M = gap2 and mitosis) of the cell cycle compared by One-way analysis of variance (ANOVA), followed by the Tukey *post hoc* test. The different letters (i.e., A, B, C, D, E, F) indicate statistical differences in each treatment at *p* < 0.05. (C) Monolayer culture pattern of RAW macrophages microscopic optic analysis (×40, scale bar = 20 μm) in control (C) cells and cells exposed to OLZ and PHA. No-activated C-cells presented higher frequency of spheric cells typical of monocytes. Cells PHA and OLZ exposed presenting a macrophage spreading pattern that is observed in inflammatory-activated cells.

From this first analysis, OLZ at 0.03 μg/mL concentration was chosen to conduct complementary protocols, since this concentration increased cell proliferation and was into the plasmatic therapeutic range of this drug. Complementary analysis was performed to confirm that this concentration could trigger macrophage activation by evaluation of cell cycle modulation and cytomorphological macrophage patterns in 72h cell cultures (Fig 2). Macrophages OLZ-exposed presented higher frequency of S-phase cells than C-group. This effect was higher than cells just PHA-exposed, that is a natural antigen used to trigger *in vitro* macrophage inflammatory activation. OLZ-exposure also increased frequency of G2/M cells than C-group, whereas cells PHA-exposed presented significant lower frequency of cells in this phase than C-group. Both, PHA and OLZ caused macrophage spreading pattern in monolayer cultures that indicate an inflammatory state.

Further, RAW cells were concomitantly exposed with OLZ at this concentration and Li at 0.7 mEq/L concentration, an anti-inflammatory drug (Fig 2A). All treatments increased cellular proliferation compared to the control group and proliferation was highest in cells exposed to OLZ plus Li. Modulation of oxidative markers that are involved with inflammatory processes were also evaluated (Fig 2B). All treatments increased SA and ROS levels than C group. However, this effect was more pronunciated in cells just OLZ- treated. Therefore, it seems that Li attenuated OLZ oxidative effects on macrophage cells. Just cells OLZ-exposed presented higher NO levels than C group, including cells concomitantly OLZ and Li-treated.

**Fig 3:**
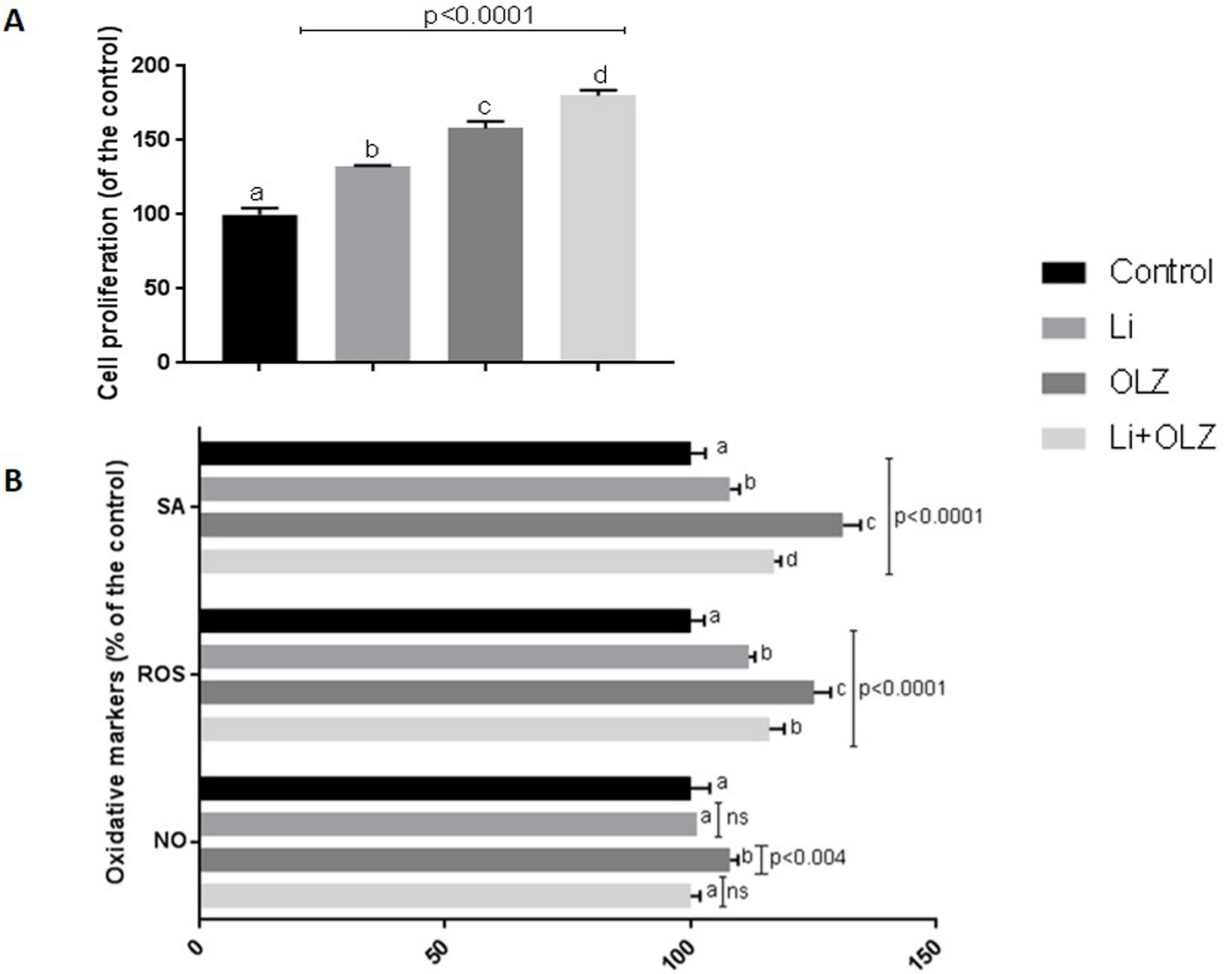
Interaction between OLZ (0.03 μg/mL) and Li (0.7 mEq/L) on proliferation (A) and modulation of oxidative markers superoxide anion (SA), reactive oxygen species (ROS), and nitric oxide (NO) (B). Treatments were compared by one-way analysis of variance (ANOVA), followed by the Tukey post hoc test. The different letters (i.e., A, B, C, D) indicate statistical differences in each treatment at p < 0.05. Treatments identified with A-letter were statistically s imilar to untreated-cells group (C).

**Fig 4:**
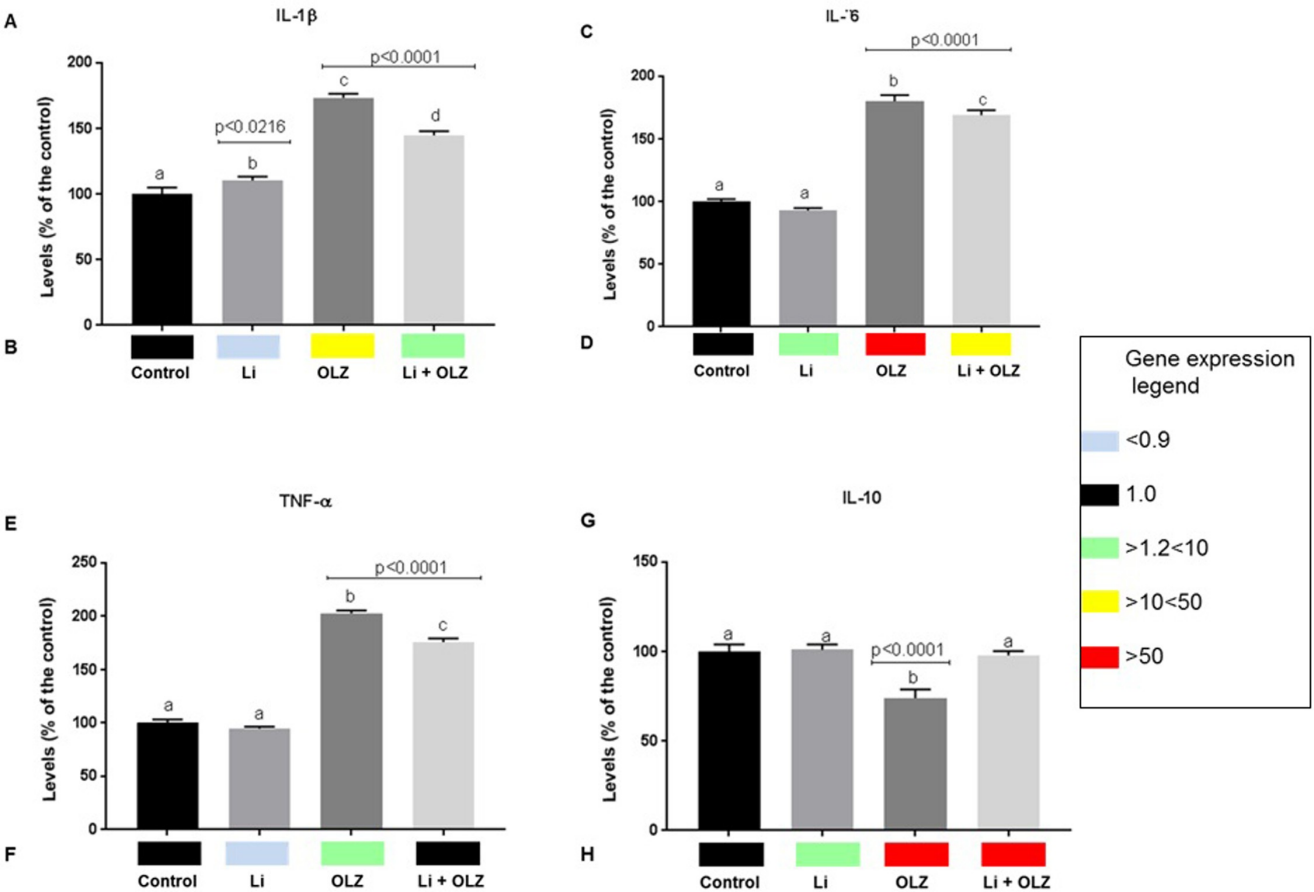
Modulation of protein and gene expression cytokines (IL-1β, IL-6, TNFα, IL-10) involved with inflammatory response of RAW macrophages exposed to OLZ (OLZ, 0.03 μg/mL) and Li (Li, and 0.7 mEq/L). Treatments were compared by one-way analysis of variance (ANOVA), followed by the Tukey post hoc test. The different letters (i.e., A, B, C, D) indicate statistical differences in each treatment at *p* < 0.05. Treatments with A letter were considered with values similar to C untreated-cells group. Gene expression of each cytokine in each treatment is represented by colored squares (black = gene expression similar to C-group; green square = gene overexpression ≥ 1.2 < 10 times than C-group; yellow square = gene overexpression ≥ 10 < 50 times than C-group; red square = gene overexpression ≥ 50 times than C-group. The beta-actin housekeeping was used as internal control to normalized gene expression analysis among treatments.

Analysis of gene and protein expression of four inflammatory cytokine markers among treatments was also performed and results are presented in Fig 3. Cells just OLZ treated presented higher protein levels of IL-β, IL-6 and TNFα proinflammatory cytokines (Fig 4). On the other hand, cells on this treatment presented lower levels of IL-10, an anti-inflammatory cytokine. OLZ induced gene overexpression of IL-β, IL-6 and IL-10 cytokines. Therefore, the role of results indicated OLZ proinflammatory effect on RAW cells.

At contrary, cells just Li-exposed presented similar levels of IL-6, TNF-α and IL-10 cytokines than C non-activated macrophages group. However, a slight, but significant increase of IL- IL-β was observed in cells just Li-exposed t than C-group. Despite these results, IL- β, as well as TNF-α gene expression were downregulated by Li-exposure. Both IL-6 and IL-10 genes were overexpressed in cells just Li-exposed.

Interaction between OLZ and Li showed a decrease on IL-β, IL-6 and TNFα protein levels than cells just OLZ-exposed. However, levels of these cytokines were still significantly higher than C-group. Despite, IL-β and IL-6 genes to be overexpressed in cells concomitantly OLZ and Li exposed, this effect was more attenuated in comparison with cells just OLZ-exposed. In the presence Li, cells OLZ exposed presented similar TNFα gene expression than control group. IL-10 gene was also overexpressed in this treatment than C-group.

## Discussion

The present *in vitro* study evaluated the potential anti-inflammatory action of Li on proinflammatory response in macrophage cells triggered by the antipsychotic drug OLZ. The results suggest that Li may partially reverse the inflammatory response caused by OLZ on macrophage cells. Based on these general results it is important to consider theoretical and methodological limitations.

In the first analysis performed here an OLZ curve-concentration showed a hormetic effect of this antipsychotic drug on macrophages. In fact, concentrations in to plasmatic therapeutic range of OLZ showed increase on macrophage proliferation, whereas concentrations > 1 μG/mL decreased significantly cellular proliferation than C-group. These results could indicate potential cytotoxic or immunosuppressive effect of OLZ on macrophages cells. Actually, previous studies reported potential apoptosis induction of OLZ on hamster pancreatic β cell line with marked apoptotic events on these cells. This action could explain potential pro-diabetic effect associate with OLZ administration [31]. Pessina et al [32] also described potential cytotoxic effect of high OLZ concentration on macrophages. However, main focus of the present study was clarifying potential interaction between OLZ and Li, that has some anti-inflammatory properties. For this reason, complementary analysis involving analysis of OLZ on apoptosis modulation was not performed here. Perhaps, complementary studies about this issue could be performed, since some recent investigations, such as performed by Sanomachi et al [33] have described potential beneficial OLZ effect on cancer patients by down-regulation of survivin, which has been implicated in multidrug chemoresistance and apoptosis induction.

Investigations of OLZ effects on inflammatory modulation are relatively incipient. In RAW 264.7 macrophage cells we found a potential important hormetic effect on inflammatory response observed by cellular proliferation rates (Fig 1). This hormetic OLZ effect could explain differences between results found here and other studies published recently in the literature, such as performed by Stapel et al [34]. These authors showed that when human peripheral blood mononuclear cells (PBMCs) obtained from healthy adults were *in vitro* exposed to 10^−4^ M OLZ occurred decreasing in mRNA and protein levels of IL-1β, IL-6, and TNF-α than control group, at 72 h cell cultures. However, the OLZ concentration (31.24 μg/mL) used by authors was thousand times greater than concentration tested here (0.03 μg/mL), that is into therapeutic plasmatic OLZ concentration range [24]. In fact, we think that in an *in vitro* protocol the use of OLZ at a plasmatic therapeutic concentration could be more realistic considering pharmacokinetics of this drug. Into the body, OLZ is metabolized by cytochrome P450 and more than 40% of the oral doses is removed by the hepatic first-pass effect. In these terms, it is not expected that high OLZ concentrations arrive in peripheral body cells including macrophages.

Previous evidence suggested that immune system alteration associated with OLZ treatment may contribute to antipsychotic-induced weight gain [35]. Inflammatory effects of OLZ have not been well-characterized. However, in light of recent findings that OLZ may induce inflammatory signaling, it is possible that concomitant use of OLZ with anti-inflammatory drugs may attenuate proinflammatory effects of OLZ. In psychiatric practice use of Li, which exerts anti-inflammatory effects via inhibitor of GSK-3, is common [36].

Studies examining the beneficial effects of Li in combination with OLZ may help to understand the increase in efficacy of treatment of some psychiatric symptoms conferred by combination therapies. Clinical studies have demonstrated that concomitant treatment with OLZ and Li reduced rehospitalization risk after a manic episode [20,37]. Moreover, previous studies have shown that coadministration of melatonin with OLZ and Li in adolescents with Bipolar Disorder may reduce weight gain associated with OLZ treatment [38]. The beneficial effects of coadministration demonstrated in these studies provided justification for our evaluation of effects of OLZ and Li on oxidative and inflammatory metabolism.

Evaluation of *in vitro* interactions of OLZ and Li was carried out by treatment of cells at concentrations in the recommended therapeutic plasma range. The range evaluated for OLZ was 0.02 μg/mL to 0.08 μg/mL according to guidelines of drug therapeutic monitoring [39]. The therapeutic Li concentration used was 0.07 mEq/L, which has been previously evaluated in *vitro* [21,22]. Therefore, results of this study may be representative of interactive *in vivo* effects between OLZ and Li.

Previous studies have consistently supported the hypothesis that oxi-inflammatory effects of OLZ administration are associated with metabolic side effects [13,16,18]. In addition, anti-inflammatory properties of Li are also well-characterized and seem to contribute to reduction in frequency of manic episodes in bipolar patients [35,40]. However, potential effects of interaction between OLZ and Li on metabolic side effects are not clear. For example, the study performed by Katagiri et al. [41] suggested no direct impact of Li treatment on OLZ side effects. In contrast, we demonstrated that cotreatment with OLZ and Li resulted in decreased proinflammatory cytokines and increased of levels of IL-10, an anti-inflammatory cytokine.

Our results are placed in appropriate context by considering functional aspects of macrophages in relation to inflammatory response. Macrophages present two distinct phenotypic subtypes: classically and alternatively activated macrophages. Classically activated, or M1, macrophages are pro-inflammatory and polarized by antigens such as lipopolysaccharide (LPS) and PHA, resulting in production of pro-inflammatory cytokines such as interleukin-1β (IL-1β), IL-6, IL-12, IL-23, and TNF-α. Conversely, alternatively activated, or M2, macrophages are anti-inflammatory and immunoregulatory cells polarized by Th2 cytokines such as IL-4 and IL-13. M2 cells also produce anti-inflammatory cytokines such as IL-10 and TGF-β. The role of M1/M2 macrophage polarization balance in organ response to inflammation or injury has been well-established [42].

When inflammation is triggered macrophages first exhibit the M1 phenotype, releasing mainly IL-1β, IL-6, TNF-α, and IL-12 in response. As the M1 phase continues, damage may occur. Higher levels of proinflammatory cytokines indicate a more intense inflammatory response by M1 macrophages. Conversion to the M2 phenotype associated with increased release of anti-inflammatory cytokines such as IL-10 is crucial in resolution of the proinflammatory state. M2 macrophages are able to suppress inflammation, contributing to tissue repair, remodeling, vasculogenesis, and maintenance of homeostasis [42].

Activated macrophages cultured for 72 h exposed to a single antigen were expected to produce higher levels of proinflammatory cytokines. However, we also observed increased anti-inflammatory cytokines. Anti-inflammatory cytokines produced by activated macrophages may influence macrophage inflammatory response in three ways: (1) M1 phenotype suppression in the presence of an antigen molecule; (2) decreased production of proinflammatory cytokines; (3) faster conversion from M1 to M2 phenotype [42].

As cytokine protein and gene expression are the best markers for determination of macrophage polarization, our results showed that M1 polarization of RAW 264.7 macrophages were greatly attenuated by Li treatment, as levels of most cytokines remained similar to the untreated group. In contrast, clear M1 polarization was observed in OLZ-treated macrophages, and this inflammatory response was partially attenuated by administration of Li. These results support the hypothesis that Li may attenuate the inflammatory cascade triggered by the antipsychotic drug OLZ.

Of note, macrophages treated with only Li showed greater cellular proliferation and slightly higher IL-1β levels than control. Based on previous studies, slight macrophage activation may occur in response to Li because the RAW 264.7 macrophage cell line is very sensitive to chemical changes in culture medium [43]. Therefore, these results may represent a limitation of this *in vitro* model rather than a true effect of Li on macrophage activation.

Our results also showed an effect of cotreatment with Li and OLZ on oxidative metabolism markers associated with the inflammatory process. Cells exposed only to OLZ produced higher levels of superoxide, ROS, and NO. However, Li attenuated levels OLZ-induced oxidative markers. Of particular interest is the effect of OLZ treatment on NO levels, as NO is an important pleiotropic signaling molecule. NO induces concentration-dependent enhancement of endothelial cell proliferation, angiogenesis, and acceleration of wound healing. Conversely, NO is an oxidative molecule, and may also contribute to induce inflammation [44].

In addition to increased production of pro-inflammatory cytokines, M1 macrophages produce increased NO via inducible nitric oxide synthase (iNOS) [44]. In contrast, M2 macrophages are characterized by expression of the enzyme arginase, which hydrolyzes arginine to ornithine and urea. This reaction limits arginine availability for NO synthesis [45]. Therefore, analysis of NO levels may indicate phenotype of macrophage cells. Cotreatment with OLZ and Li significantly decreased NO levels, supporting the hypothesis that Li may attenuate the proinflammatory effect triggered by OLZ.

Decreased ROS levels in macrophages in response to cotreatment with OLZ and Li highlights the benefit of this interaction on the inflammatory pathway since accumulating evidence supports the hypothesis that redox signaling plays a role in macrophage polarization [46]. Moreover, elevation of ROS by OLZ results from changes in mitochondrial functioning suggesting that Li may act on mitochondria. However, this presumption is still speculative since we did perform direct assays of potential mitochondrial dysfunction triggered by OLZ exposure. Despite this methodological limitation, it is important to point out that some previous studies have suggested that antipsychotic effects on metabolism may be associated with alteration of mitochondrial function. This alteration, associated with a chronic inflammatory state, may contribute to development of metabolic syndrome in patients that use antipsychotic drugs [47, 48].

This presumption is corroborated by a recent study performed by Scani et al [49] using peripheral blood mononuclear cells (PBMCs) from schizophrenic patients exposed to antipsychotic drugs. Results from this study showed that these drugs may induce mitochondrial dysfunction. Mitochondria are key organelles responsible for energy production and control many processes from signaling to cell death. The function of the mitochondrial electron transport chain is coupled with production of ROS in the form of superoxide anion and hydrogen peroxide. Mitochondrial ROS overproduction and changes in mitochondrial redox homeostasis are involved in a number of neurological and somatic conditions [50].

As OLZ treatment of macrophages increased superoxide and ROS, which includes hydrogen peroxide, these results indirectly suggest that this drug may cause mitochondrial dysfunction. Attenuation of ROS production by Li exposure may be possible as described in an investigation performed by Kim et al [51]. This study demonstrated that mitochondrial dysfunction and lipid peroxidation in rat frontal cortex triggered by chronic NMDA administration was partially reversed by Li treatment. In humans, Souza et al [52] showed that BD patients treated for six weeks with Li had reduced mitochondrial dysfunction in Complex 1 of the electron transport chain.

Despite limitations and constraints related to *in vitro* studies, our results highlight potential for use of Li as an additional therapeutic approach in the prevention of oxidative and inflammatory effects triggered by OLZ. However, further studies are needed to clarify the mechanisms involved in the protective effect of Li on OLZ-induced inflammatory response and the impact Li may have on the prevention of metabolic syndrome in patients treated with second generation antipsychotics drugs.

## Conclusion

Concomitant *in vitro* macrophage exposure to OLZ and Li suggests that Li may attenuate oxidation and inflammation. It can be inferred that concomitant use of OLZ may contribute to attenuation of some side effects triggered by OLZ administration.
